# Symptomatic Premature Ventricular Contractions in the Context of Cardiac Memory Following Septal Accessory Pathway Ablation: A Case Report

**DOI:** 10.1155/cric/1241417

**Published:** 2025-12-22

**Authors:** Manuel J. Vogel, Jonas Herting, Moritz T. Huttelmaier, Thomas H. Fischer

**Affiliations:** ^1^ Department of Internal Medicine I, University of Wuerzburg, University Clinic, Wuerzburg, Germany, uni-wuerzburg.de

## Abstract

**Introduction:**

Wolff–Parkinson–White (WPW) syndrome is a congenital heart disorder marked by an accessory electrical pathway (AP) causing reentrant tachycardias. This report presents a case of WPW successfully treated with catheter ablation but followed by transient symptomatic premature ventricular contractions (PVCs).

**Case Summary:**

A 27‐year‐old WPW patient was referred due to paroxysmal tachycardias. The baseline electrocardiogram confirmed ventricular pre‐excitation, and the patient underwent radiofrequency ablation targeting a right septal AP. Some days later, the patient experienced palpitations distinct from preablation symptoms. A 24‐h Holter ECG and exercise testing revealed frequent, monomorphic PVCs originating from the previously pre‐excited region. Low‐dose beta‐blocker therapy alleviated symptoms, and there was no evidence of PVC recurrence after tapering temporary beta‐blocker medication.

**Discussion:**

This case illustrates a new onset of PVC after ablation of septal AP in WPW syndrome. We postulate that transient electric instability due to AP‐associated cardiac memory led to enhanced local automatic activity resulting in PVC.

## 1. Introduction

Wolff–Parkinson–White (WPW) syndrome is a congenital cardiac disorder that is characterized by the presence of an accessory electrical pathway (AP) in the heart. This pathway leads to altered conduction that circumvents the typical route through the atrioventricular (AV) node, which predisposes to reentrant tachycardias and carries a significant risk of rapid ventricular conduction in atrial fibrillation.

Catheter ablation is regarded as the preferred treatment option for WPW patients who are symptomatic or at higher risk of sudden cardiac death due to atrial fibrillation with rapid ventricular response [1]. The procedure is aimed at exactly localizing the AP via electrophysiologic study and electroanatomic mapping and ablating the accessory pathway through the application of thermal energy. The success rate of catheter ablation in patients with WPW syndrome is high (up to 95%) with a low risk of complications [2]. We present an unusual case of a patient who newly developed premature ventricular contractions (PVCs) following AP ablation.

## 2. Case Presentation

**Figure 1:**
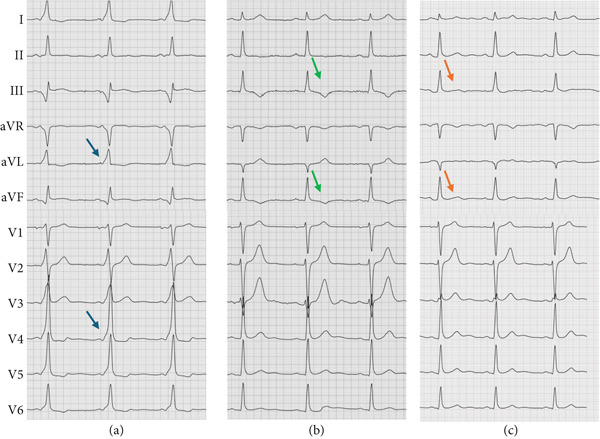
Twelve‐lead resting ECG (50 mm/s, 10 mm/mV) (a) before, (b) 1 day, and (c) several weeks after RF ablation. (a) Pre‐excitation with a shortened PQ interval (100 ms) and a distinct delta wave (blue arrows, positive in I, aVL, and V2–V6; negative in III and aVF) indicating a (postero‐)septal AP. (b) No discernible pre‐excitation after RF ablation but new T‐wave inversions in III and aVF (green arrows). (c) T‐wave abnormalities in III and aVF are only barely detectable (orange arrows).

**Figure 2:**
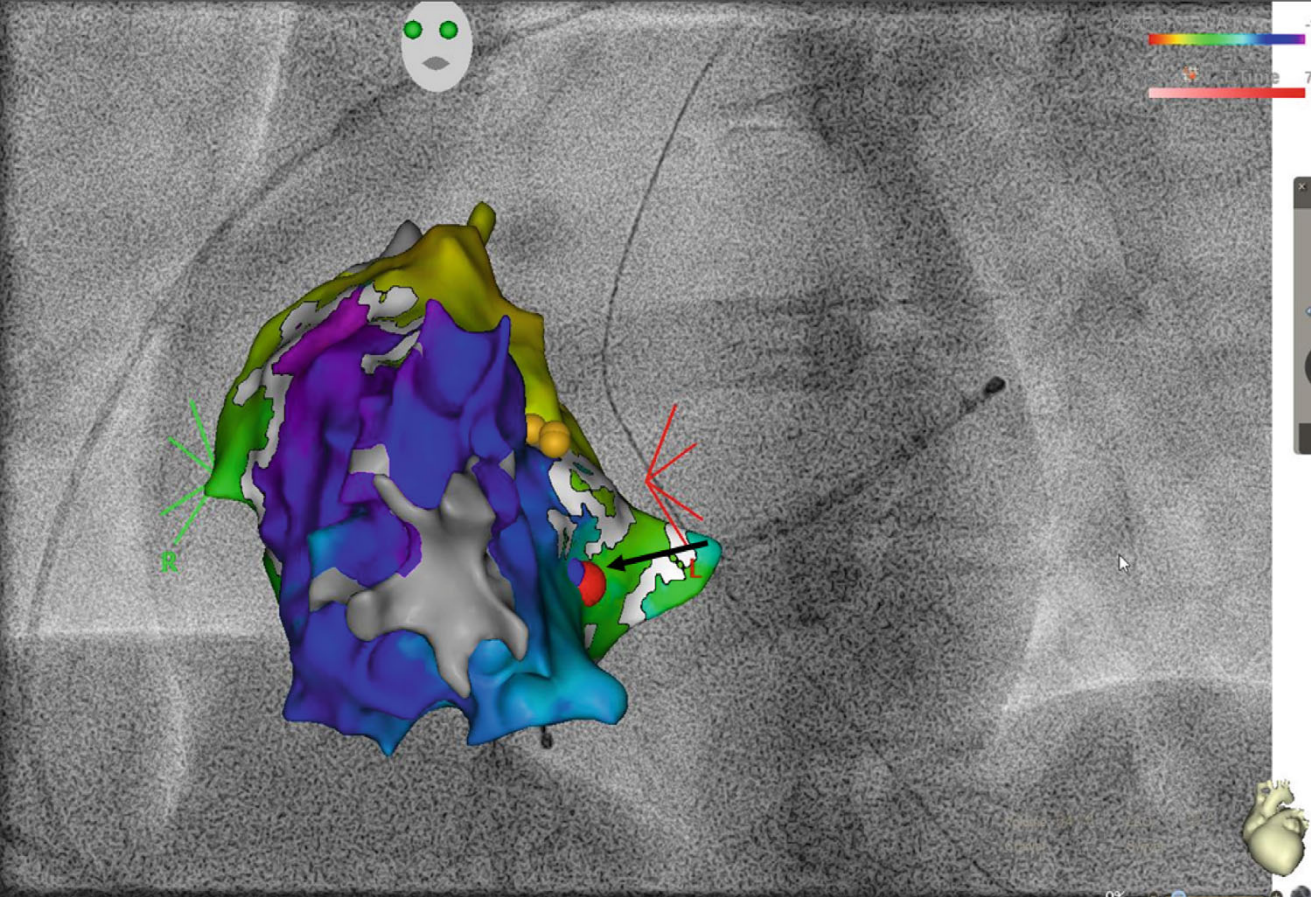
3D electroanatomical (“open‐window”) mapping (Biosense Webster CARTO 3) and fluoroscopic projection (LAO 45°) of the right atrium and ventricle acquired with a multipolar catheter (PENTARAY NAV ECO) revealing an accessory pathway in the posterior septum (marked by a black arrow). Yellow spheres: His signal; red sphere: site of RF ablation.

**Figure 3:**
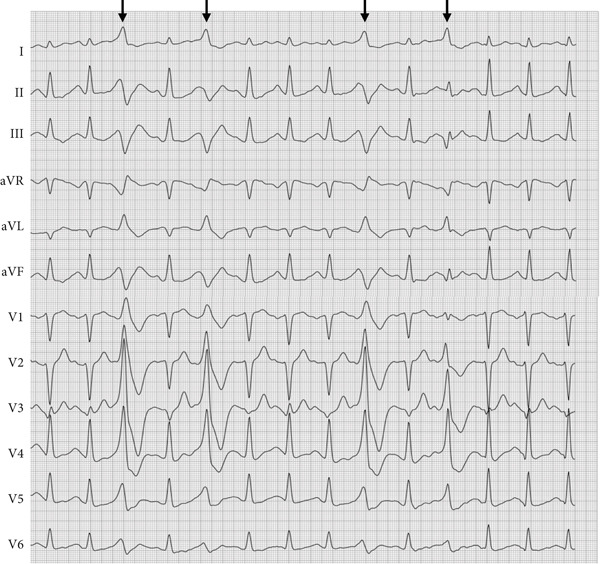
ECG (50 mm/s, 10 mm/mV) during bicycle exercise at 125 W. PVC (black arrows) with broad QRS (approximately 150 ms); superior QRS axis, positive QRS in Leads I and aVL and positive concordance in Leads V1–V6 point toward a basal posteroseptal exit.

A 27‐year‐old male is presented to the rhythm clinic with WPW syndrome characterized by paroxysmal tachycardias and dizziness. Upon presentation, the electrocardiogram (ECG) demonstrated ventricular pre‐excitation with a shortened PQ interval (100 ms) and QRS widening (140 ms) due to a distinct delta wave (positive in Leads I, aVL, and V2–V6; negative in Leads III and aVF; Figure 1a), indicating a (postero‐)septal AP. Thus, the indication for an electrophysiological study was given aiming at locating and ablating the AP. The electrophysiological study with 3D electroanatomical open‐window mapping (Biosense Webster Carto 3) of the right atrium and right ventricle revealed the presence of a right septal AP in the posterior triangle of Koch (Figure 2). Radiofrequency (RF) ablation (40 W, 60 s) was performed at the atrial aspect of the identified area of the earliest ventricular activation with sufficient distance to the solid AV node. Pre‐excitation in the surface ECG was lost after 5 s of ablation and did not recur within 30 min of waiting time after ablation, even following catecholaminergic stimulation with isoprenaline. One day after RF ablation, the surface ECG demonstrated the absence of pre‐excitation but revealed T‐wave inversions in Leads III and aVF (Figure 1b). The patient was discharged free of symptoms. Some days later, however, the patient was again conferred to our rhythm clinic due to the recurrence of palpitations, which were characterized as a stumbling heartbeat sensation, particularly during periods of physical exertion. The 12‐lead resting ECG initially demonstrated no abnormal findings. To further clarify the symptoms, a 24‐h Holter ECG and treadmill testing were performed, which revealed increased monomorphic PVC with a broad ventricular complex (QRS duration approximately 150 ms, Figure 3). The PVC exhibited a positive ventricular complex in Leads I and aVL, a summated isoelectric vector in Leads II and III, and positive concordance in Leads V1–V6. Preceding P waves could not be identified. As a differential diagnosis, intermittent AV conduction via a persisting AP had to be considered, particularly in view of the patient′s history. Therefore, an adenosine test was conducted. The administration of 18‐mg intravenous adenosine resulted in a notable prolongation of the AV interval, which proved physiological AV conduction properties and excluded persisting pre‐excitation. As a result, a diagnosis of symptomatic PVC was rendered. Surface ECG analysis revealed a basal posteroseptal exit of the PVC, indicating an origin near the previously pre‐excited region. Transthoracic echocardiography demonstrated unremarkable results. The administration of low‐dose beta‐blocker therapy with bisoprolol 1.25 mg twice daily resulted in a notable improvement in symptoms. The beta‐blocker therapy was discontinued a few weeks later, and no further episodes of palpitations or increased PVC burden were observed. Also, the repolarization disturbances observed in the inferior leads following the ablation were only barely detectable (Figure 1c).

## 3. Discussion

This case report highlights the occurrence of transient symptomatic PVC in a 27‐year‐old male with WPW syndrome after RF ablation of a right septal AP. Post‐ablation, the disappearance of pre‐excitation confirmed successful disruption of the AP. However, surface ECG revealed conduction disturbances consisting of preterminal negative T‐waves in inferior leads. At the time of discharge, the patient was free of symptoms. However, within several days, the patient experienced recurrent palpitations, distinct from his preablation symptoms. Further investigation, including a 24‐h Holter ECG and exercise testing, revealed the presence of frequent monomorphic PVC originating from the basal posteroseptal region in direct proximity to the previously pre‐excited myocardium. One potential hypothesis is that the ablation procedure itself causes localized tissue damage and inflammation, which may lead to electrical instability of the myocardium [3, 4]. However, in the case of ablation of the postero‐septal AP, the primary RF lesion is situated within the right atrium. It is therefore unlikely that local myocardial injury is the cause of the patient′s PVC. The localization of the PVC rather suggests that the ectopic activity is related to the local repolarization abnormalities observed after ablation that is commonly referred to as cardiac memory. The phenomenon of cardiac memory is frequently seen following AP ablation, manifesting as T‐wave inversion, particularly in inferior leads [5]. Interestingly, a right posteroseptal pathway has been identified as a primary predictor of cardiac memory [5]. Earlier research demonstrated that the altered ventricular activation through the AP leads to changes in local ion channel expression and conductance properties, especially through a reduction of repolarizing potassium channels (IKr) in the epicardium [5–8]. As a consequence, left ventricular repolarization gradients rise and diminish the repolarization reserve, which can lead to QT prolongation and even QT‐related ventricular arrhythmias (torsade de pointes) [7]. When the normal ventricular activation is restored after AP ablation, myocardial changes undergo a gradual recovery [6, 7]. This may potentially lead to intermittent electric instability with enhanced local automatic activity and the occurrence of PVC. During a period of several weeks or even months, in which transcriptional reprogramming and remodeling of ion channels are ongoing, repolarization disturbances and electric instability temporarily persist [6, 7]. Furthermore, prior research demonstrated that RF ablation damages local parasympathetic fibers, which occur in high density in the septal region [9, 10]. Thus, changes in autonomic tone, particularly during periods of increased sympathetic activity such as exercise, may have contributed to the occurrence of PVC in our patient by exacerbating the cardiac memory–induced electric instability and repolarization disturbances. The available data on PVC following AP ablation are limited. Mujovic et al. investigated new arrhythmias following AP ablation and reported on two cases of septal AP with PVC with exit near the previously pre‐excited region at least 1 month after the procedure. Furthermore, the septal location of the AP was identified as an independent predictor of new arrhythmias [11]. Bera et al. reported on a patient with new PVC after ablation of a septal pathway, which ceased spontaneously after a few months [12]. This case report supports the idea of transient alterations in ectopic activity. In our patient, the PVC were addressed by a low‐dose beta‐blocker therapy leading to a notable improvement in symptoms. A few weeks later, the medication was discontinued, and the patient did not experience a recurrence of palpitations or arrhythmias. We postulate that as soon as the myocardium recovered from the cardiac memory the ectopic activity subsided and the patient therefore no longer required antiarrhythmic medication.

## 4. Take‐Home Messages

Cardiac memory after ablation of an accessory pathway can lead to ventricular arrhythmias such as PVCs. These arrhythmias are typically transient in nature and could be effectively treated with a beta‐blocker in this case.

## Ethics Statement

The authors confirm that written consent for submission and publication of this case report including images and associated text has been obtained from the patient in line with COPE guidance. The patient′s treatment and follow‐up care were carried out in accordance with guidelines and standard operating procedures.

## Conflicts of Interest

The authors declare no conflicts of interest.

## Funding

T.H.F. is funded by the German Heart Research Foundation (Deutsche Stiftung für Herzforschung) and the DFG (Deutsche Forschungsgemeinschaft). J.H. and M.T.H. were funded by a clinician–scientist scholarship of the Interdisciplinary Center for Clinical Science at the University of Würzburg (IZKF). Open access funding is enabled and organized by Projekt DEAL.

## Data Availability

The data that support the findings of this study are available on request from the corresponding author. The data are not publicly available due to privacy or ethical restrictions.

## References

[1] Brugada J. , Katritsis D. G. , Arbelo E. , Arribas F. , Bax J. J. , Blomstrom-Lundqvist C. et al., 2019 ESC Guidelines for the Management of Patients With Supraventricular tachycardiaThe Task Force for the management of patients with supraventricular Tachycardia of the European Society of Cardiology (ESC), European Heart Journal. (2020) 41, no. 5, 655–720, 10.1093/eurheartj/ehz467, 31504425. 31504425

[2] Jackman W. M. , Xunzhang W. , and Friday K. J. , Catheter Ablation of Accessory Atrioventricular Pathways (Wolff-Parkinson-White Syndrome) by Radiofrequency Current, NEJM. (1991) 324, no. 23, 1605–1611, 10.1056/NEJM199106063242301, 2-s2.0-0025870671. 2030716

[3] Osmancik P. , Bacova B. , Hozman M. , Pistkova J. , Kunstatova V. , Sochorova V. , Waldauf P. , Hassouna S. , Karch J. , Vesela J. , Poviser L. , Znojilova L. , Filipcova V. , Benesova K. , and Herman D. , Myocardial Damage, Inflammation, Coagulation, and Platelet Activity During Catheter Ablation Using Radiofrequency and Pulsed-Field Energy, JACC: Clinical Electrophysiology. (2024) 10, no. 3, 463–474, 10.1016/j.jacep.2023.11.001, 38085214. 38085214

[4] Liu D. , Li Y. , and Zhao Q. , Effects of Inflammatory Cell Death Caused by Catheter Ablation on Atrial Fibrillation, Journal of Inflammation Research. (2023) 16, 3491–3508, 10.2147/JIR.S422002, 37608882. 37608882 PMC10441646

[5] Austin K. M. , Alexander M. E. , and Triedman J. K. , Pediatric T-Wave Memory After Accessory Pathway Ablation in Wolff-Parkinson-White Syndrome, Heart Rhythm. (2022) 19, no. 3, 459–465, 10.1016/j.hrthm.2021.11.007, 34767987. 34767987 PMC9026902

[6] Ghosh S. , Rhee E. K. , Avari J. N. , Woodard P. K. , and Rudy Y. , Cardiac Memory in Patients With Wolff-Parkinson-White Syndrome: Noninvasive Imaging of Activation and Repolarization Before and After Catheter Ablation, Circulation. (2008) 118, no. 9, 907–915, 10.1161/CIRCULATIONAHA.108.781658, 2-s2.0-52049123827, 18697818. 18697818 PMC2747627

[7] Viskin S. , Chorin E. , Schwartz A. L. , Kukla P. , and Rosso R. , Arrhythmogenic Effects of Cardiac Memory, Circulation. (2022) 146, no. 15, 1170–1181, 10.1161/CIRCULATIONAHA.122.061259, 36214133. 36214133

[8] Takada Y. , Akahoshi M. , Shibata Y. , Shimizu A. , Yoshida Y. , Yamada T. et al., Changes in Repolarization Properties With Long-Term Cardiac Memory Modify Dispersion of Repolarization in Patients With Wolff-Parkinson-White Syndrome, Journal of Cardiovascular Electrophysiology. (2002) 13, no. 4, 324–330, 10.1046/j.1540-8167.2002.00324.x, 2-s2.0-0036105585, 12033346. 12033346

[9] Jinbo Y. , Kobayashi Y. , Miyata A. , Chiyoda K. , Nakagawa H. , Tanno K. , Kurano K. , Kikushima S. , Baba T. , and Katagiri T. , Decreasing Parasympathetic Tone Activity and Proarrhythmic Effect After Radiofrequency Catheter Ablation, Japanese Circulation Journal. (1998) 62, no. 10, 733–740, 10.1253/jcj.62.733, 2-s2.0-0031716668, 9805253. 9805253

[10] Kocovic D. Z. , Harada T. , Shea J. B. , Soroff D. , and Friedman P. L. , Alterations of Heart Rate and of Heart Rate Variability After Radiofrequency Catheter Ablation of Supraventricular Tachycardia Delineation of Parasympathetic Pathways in the Human Heart, Circulation. (1993) 88, 4 pt. 1, 1671–1681, 8403312. 8403312 10.1161/01.cir.88.4.1671

[11] Mujović N. , Grujić M. , Mrdja S. , Kocijancić A. , and Mujović N. , The Occurrence of New Arrhythmias After Catheter-Ablation of Accessory Pathway: Delayed Arrhythmic Side-Effect of Curative Radiofrequency Lesion?, Srpski Arhiv za Celokupno Lekarstvo. (2011) 139, no. 7–8, 458–464, 10.2298/SARH1108458M, 2-s2.0-81055123600, 21980654. 21980654

[12] Bera D. , Majumder S. , Sarkar R. , Kar A. , and Mukherjee S. S. , Arrhythmogenicity of Radiofrequency Ablation: Symptomatic Non-Sustained Ventricular Tachycardia and Frequent Premature Ventricular Ectopics After Accessory Pathway Ablation, Indian Pacing and Electrophysiology Journal. (2021) 21, no. 2, 120–123, 10.1016/j.ipej.2020.11.018, 33246079. 33246079 PMC7952768

